# Cysteine string protein α and a link between rare and common neurodegenerative dementias

**DOI:** 10.1038/s44400-025-00016-0

**Published:** 2025-07-03

**Authors:** Matthew J. Rosene, Bruno A. Benitez

**Affiliations:** Department of Neurology, Beth Israel Deaconess Medical Center, Harvard Medical School, Boston, MA USA

**Keywords:** Cellular neuroscience, Molecular neuroscience, Alzheimer's disease, Amyotrophic lateral sclerosis, Dementia, Motor neuron disease, Neurodegeneration, Parkinson's disease

## Abstract

The maintenance of protein homeostasis and overall protein quality control dysfunction are associated with dementia. Cysteine string protein α (CSPα) is an endolysosomal cochaperone that facilitates the fusion of secretory and synaptic vesicles to the cell membrane. CSPα interacts with multiple proteins related to the proteostasis network and exocytic pathways and is often dysfunctional in synaptopathies. Since the initial discovery of CSPα 30 years ago, subsequent research has demonstrated a protective role of CSPα, especially in synaptic maintenance. However, the discovery of heterozygous CSPα mutations in 2011 causing adult-onset neuronal ceroid lipofuscinosis (ANCL) shifted the back-then prevalent dogma of unique synaptic function to include an endolysosomal role for CSPα. Recently, CSPα has been involved in the exocytosis of aggregate-prone proteins through either the misfolding-associated protein secretion (MAPS) or unconventional secretory pathways linking the molecular mechanism of rare and common neurodegenerative diseases. Here, we propose a novel molecular and pathophysiological model of CSPα-associated dementia, outline the increasing evidence of a broader role of CSPα in neurodegeneration, propose the role of CSPα in the synaptic secretion of neurodegenerative-associated proteins, and discuss the modulation of CSPα as a molecular target for common dementias.

## Proteostasis: focus on molecular chaperones

Proteostasis involves sophisticated protein synthesis, quality control, and degradation^1^ processes. Addressing defective proteins promptly and efficiently is crucial for maintaining cellular homeostasis^1^. There are multiple protein quality control (PQC) pathways that correct for unfolded or misfolded proteins. These include the unfolded protein response (UPR) in the endoplasmic reticulum (ER)^2–4^, the heat shock response (HSR), and the degradation of misfolded and aggregated proteins through the ubiquitin-proteasome system (UPS) or the autophagy-lysosome pathway (ALP)^1,5^. However, the internal and external stresses accompanying aging contribute to the decline in the overall capacity of the proteostasis network^1^. Furthermore, these protein aggregates can sequester essential cellular components that interfere with and hinder crucial cellular processes, deform and disrupt cellular membranes, and spread between cells as seeds to propagate the misfolding of native proteins in nearby healthy cells throughout the brain. Consistent with this, proteostasis failure plays a significant role in numerous neurodegenerative disorders characterized by dementia, including Alzheimer’s disease (AD), Parkinson’s disease (PD), Huntington’s disease (HD), and amyotrophic lateral sclerosis (ALS)^6–8^. In these diseases, misfolded or mutant variants of proteins such as tau and amyloid-β (Aβ) in AD^9^, huntingtin (HTT) in HD^10^, alpha-synuclein in PD and Lewy body dementia^11^, and TAR DNA-binding protein 43 (TDP-43), superoxide dismutase 1 (SOD1), optineurin (OPTN), fused in sarcoma (FUS), and ubiquilin-2 (UBQLN2) in ALS^12^, form protein aggregates and place increasing proteotoxic stress on the PQC pathways of the cells.

Molecular chaperones play a central role in maintaining cellular proteostasis. Heat shock proteins (HSPs) are a family of evolutionarily conserved chaperones first described in 1962 in Drosophila after exposure to high temperatures^13^. HSPs are classified according to their molecular weight into HSP10, HSP40, HSP60, HSP70, HSP90, and HSP110. There are 332 chaperone genes in the human “chaperome”, including 142 genes encoding Hsp90, Hsp70, Hsp60, Hsp40, and small HSPs^14^. Four heat shock transcription factors (HSF1-4) have been identified in the human genome, each exhibiting a unique but overlapping function^2^. HSF1 plays a central role in the cellular stress response, coordinating the transcriptional activation of genes involved in the HSR. Molecular chaperones are a diverse group of proteins that play critical roles in ATP-dependent protein folding, stability, refolding, or degradation of misfolded client proteins^15,16^. The constitutively and most widely expressed ATP-dependent molecular chaperones are 70 kDa heat shock protein (Hsp70) and 90 kDa heat shock protein (Hsp90), which work in multisubunit heterocomplexes, including cochaperones and accessory proteins. This conformation facilitates selective client interaction, the dynamic regulation of client interaction through ATPase activity modulation by cochaperones, or degradation or sequestration by the proteostasis machinery^17–19^.

**Fig. 1:**
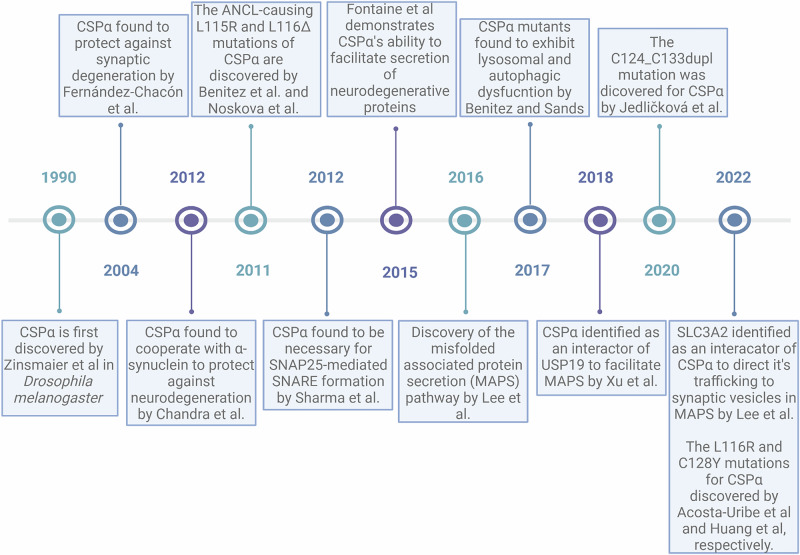
Timeline of seminal findings. Timeline summarizing seminal studies in the literature on CSPα.

This review focuses on one member, DnaJ Homolog Subfamily C Member 5 *(DNAJC5)*, of the Hsp40/J-domain family of cochaperones and its role in linking the molecular mechanism of rare and common neurodegenerative diseases. Fifty members of the Hsp40/J-domain family have been described, spanning a wide range of sizes and molecular weights (10–520 kDa)^20^. Hsp40s belong to the mammalian disaggregase machinery along with Hsp70s and the Hsp110s. Hsp40s promote protein refolding by binding and delivering misfolded clients to Hsp70 and stimulating the Hsp70 ATPase activity^21,22^. Hsp40 proteins are characterized by a 70 amino acid domain that includes the J-domain (Fig. 1). The J-domain has four α-helices with a highly conserved region called the His-Pro-Asp (HPD) tripeptide motif, which is critical for stimulating ATP hydrolysis in Hsp70^23,24^. There are subgroups of Hsp40s depending on the location of the J domain in the protein. Types I and II have the J-domain at the N-terminus. The J-domain is in a different region in type III^24,25^.

Mutations in genes encoding Hsp40/J-domain cochaperones cause genetically inherited disorders known collectively as “chaperonopathies.” Most chaperonopathies result in phenocopies of neurodegenerative diseases affecting different brain cells and regions. The basal expression levels of molecular chaperones and cochaperones vary between human tissues^26^. Interestingly, under conditions of acute cellular stress, the neuronal HSR seems less efficient than in astrocytes and microglia, which activate a fast and robust HSR^27^. Thus, it has been hypothesized that select neuronal populations exhibit low constitutive levels of HSPs and/or a high threshold of HSR induction, increasing their susceptibility to proteotoxic stress^27,28^. For example, motor neurons express higher levels of Hsc70 and Hsp27 than neurons in the substantia nigra, entorhinal cortex, and hippocampus. This higher expression is suggested to positively correlate with a higher defense capacity in these different neuronal populations^28^. Furthermore, studies in mice showed no induction of Hsp70 expression in neurons in the presence of ischemic injury or hyperthermia but did exhibit induction of expression in astroglial cells^27^. These findings suggest that cell-autonomous mechanisms maintaining proteostasis could play a role in the neurodegenerative diseases selective vulnerability or cellular resilience^27^.

Mutations in 16 Hsp40/J-domain cochaperones cause neurodegenerative diseases, including PD, peripheral neuropathy, hyperphenylalaninemia, Charcot Marie Tooth disease type II, and spastic ataxia, among others^29^. Most human diseases caused by mutations in DNAJ proteins belong to family type III (*DNAJA3, DNAJC3, DNAJC5, DNAJC6, DNAJC9, DNAJC11, DNAJC12, DNAJC13, DNAJC17, DNAJC19* and *DNAJC29*)^29^. The cause of the diverse phenotypes resulting from mutations in the DNAJC proteins and the lack of redundancy among each other are currently unknown. However, each DNAJC protein shows a different cell-type expression level, binds to specific partners, and exhibits different subcellular localizations. This is underscored by the extensive research into the role of the more prominent DnaJ family of cochaperones in neurodegeneration, spanning multiple in vitro and in vivo model systems. While many of the different DnaJ family members have been demonstrated to regulate proteopathic proteins related to AD (Tau; Aβ) (*DNAJB1; DNAJA1; DNAJB6b; DNAJC10*), PD (α-syn; Parkin) (*DNAJB1; DNAJA1; DNAJB2; DNAJB6b; DNAJB8; DNAJC10*), ALS (TDP-43; SOD1) (*DNAJB1; DNAJB2; DNAJB2a*), and Huntington’s disease (HTT) (*DNAJB6b; DNAJB1; DNAJB2; DNAJB2b; DNAJB2a; DNAJB6*), and other examples of polyglutamine (PolyQ) expansion (*DNAJB1; DNAJA1; DNAJB6b; DNAJB8; dHDJ1; dMRJ; DNAJ-1*), each family member played a different role, emphasizing the overall diversity and versatility of the DnaJ chaperone family and molecular chaperones as a whole^29^. Indeed, the literature makes clear the importance of molecular chaperones to proteostatic maintenance, and there is an urgent need better to understand them in the field of neurodegenerative research.

The *DNAJC5* gene, also known as *DNAJC5A or CLN4B*, encodes CSPα, a protein identified over 30 years ago in *Drosophila melanogaster*^30^ (Fig. 1). Its vertebrate homologs were subsequently discovered in *Torpedo californica*^31^ and rats^32^. Since its initial discovery, CSPα has been viewed primarily as a membrane-binding protein involved in regulated exocytosis^33^. Two additional CSP paralogs were discovered in vertebrates, CSP-beta (CSPβ) and CSP-gamma (CSPγ)^34^. In humans, CSPβ is encoded by the DNAJC5B gene located on chromosome 8 (ENST00000276570.10; exons: 6, coding exons: 4, transcript length: 2108 bps, translation length: 199 residues)^35–37^. DNAJC5B is expressed primarily in the testis, with the highest CNS levels being from the spinal cord^34,38^. In mice, DNAJC5B transcript levels are found in inner hair cells from the auditory epithelium, which seem to have protected them from degeneration without *DNAJC5*^39^. DNAJC5B is also expressed in mouse nerve terminals^36^. CSPγ is encoded by the human DNAJC5G gene located on chromosome 2 (ENST00000296097.8; exons: 7, coding exons: 4, transcript length: 2065 bps, translation length: 189 residues)^35,36,40^ and is found in the ER lumen^41^. Like that of DNAJC5B, the expression of DNAJC5G is limited to specific tissues, including the testis, and selected CNS regions, including the cortex and BA9 frontal cortex^34,42^. In the human brain, DNAJC5G is highly expressed in intratelencephalic cortical neurons (L2-L6 IT)^43^, which have been implicated in several neurodegenerative diseases, including PD^44^. Additional studies are needed to define the role of CSPβ and CSPγ in human diseases^35,36^.

## CSPα: the protective role in neurons and at the synapses

Establishing and maintaining synaptic transmission is paramount to neuronal health in the central nervous system (CNS) and underlies dynamic neural plasticity^45,46^. Evidence from postmortem studies strongly points to synaptic loss as the best correlate for cognitive decline in numerous neurodegenerative diseases as well as well-known dementias, including AD^47^, PD^48,49^, and HD^50^. There are multiple causes (intrinsic and extrinsic) and differences in disease-associated synaptic dysfunction, which are collectively known as synaptopathies^50^. Thus, from intrinsic processes regulating the synaptic activity, such as the expression of the crucial synaptic proteins^4^, to the extrinsic factors controlling the surrounding environment, including synaptic pruning^51^, many mechanisms underlying synaptic dysfunction in neurodegeneration remain to be elucidated. CSPα has recently been implicated in disease-specific synaptopathy roles, and CSPα-linked pathways have begun to emerge as common themes in AD, PD and HD.

CSPα was first observed within the telodendritic arborizations and synaptic terminals of neurons within the head and retina of Drosophila melanogaster with a monoclonal antibody (ab49)^30^. Drosophila deficient in the csp gene exhibited paralysis and early death along with a significant block of synaptic transmission at elevated temperatures, suggesting a possible role for CSPα in maintaining the stability of proteins in the synaptic machinery^52^. Furthermore, a Wallerian degeneration assay using olfactory receptor neurons (ORNs) in Drosophila expressing mutant DNAJC5/CSP (cspDG29203, a weak loss of function allele) showed delayed axon-synaptic degeneration, which was further enhanced in the null background (cspX1, a loss of function allele which deletes the first exon of csp; Df(3R)Exel6138, and deletion which completely removes the csp locus), suggesting that CSP is an in vivo regulator of synaptic and axonal degeneration^53^. Surprisingly, csp levels were consistently increased in degenerating synaptic fractions following injury and synapse-enriched fractions from mouse models of spinocerebellar ataxia type 5, HD, and Wallerian degeneration^53^. Additionally, in an independent Drosophila model of axonal degeneration (mutations in dAcsl and human ACSL4), vesicles and csp-positive aggregates were missorted to the lysosome^54^. However, the mechanism by which distal axons and synapses are stabilized during disease-induced degeneration is unknown. CSPα is highly conserved across several species. There is also an ortholog of the DNAJC5 in Caenorhabditis elegans, dnj-14. The dnj-14-null worms exhibited age-dependent neurodegeneration in sensory neurons, a shortened lifespan, and impaired locomotion and neurotransmission^55^.

**Fig. 2:**
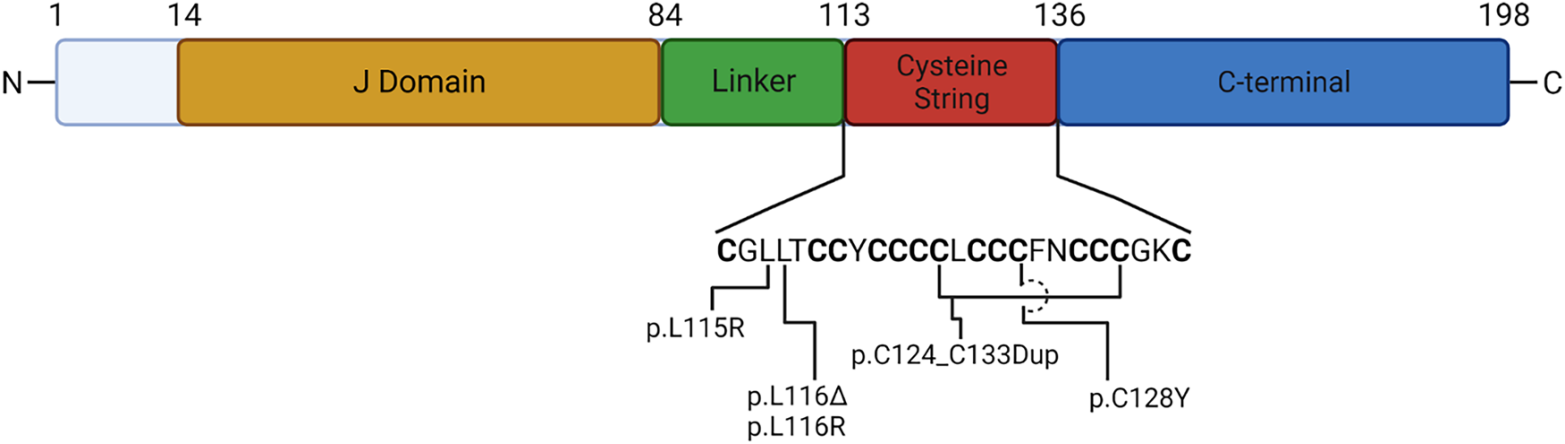
Structure of CSPα and confirmed ANCL-associated mutations. CSPα contains four separate functional domains: the J domain (aa 14–83; yellow), a linker domain (aa 84–112; green), a cysteine string domain (aa 113–135; red), and the C-terminus (aa 136–198; blue). All known mutations that have been shown to cause ANCL are located within the cysteine-string domain.

In humans, the DNAJC5 gene is located on chromosome 20 and has four coding exons (ENST00000360864.9; exons: 5, coding exons: 4, transcript length: 5249 bps, translation length: 198 residues)^56^. The 198 amino acid, 34 kDa protein comprises four distinct identified domains and exhibits chaperone activity, acts as a cochaperone with HSC70 or HSP90, and forms a trimeric complex along with small glutamine-rich tetratricopeptide repeat-containing protein (SGT) or the Rab-specific αGDP-dissociation inhibitor (Rab-αGDI)^1,57,58^. The N-terminal J domain is required for the interaction and cochaperone activity of Hsc70 and Hsp90^1,59^. The Ser10 phosphorylation in the N-terminal half of CSPα affects its role in exocytosis^60^. The J-domain seems to act through an autoinhibitory mechanism of CSPα-mediated endolysosomal translocation and protein secretion^61^. In the middle of the protein, a cysteine string domain (CSD) contains 14 densely packed cysteine residues that are highly palmitoylated by several DHHC palmitoyltransferase enzymes (DHHC3, DHHC7, DHHC15, or DHHC17). The palmitoylation of CSPα is required for membrane targeting and binding^62,63^. Ex vivo experiments demonstrated that CSPα is depalmitoylated by palmitoyl-protein thioesterase 1 (PPT1), the main intralysosomal depalmitoylase^64^. The C-terminal domain of CSPα has been implicated in protein-protein interactions (i.e., VAMP2) and homodimerization during exocytosis^35^. The extreme N-terminus (amino acids 1–13) and C-terminus are variable, whereas the other CSPα domains are highly conserved throughout multiple species^59,65^ (Fig. 2). A mis-spliced “exon 5” has been found in ALS patients and mouse models with mutations in TDP-43^66,67^, extending the C-terminal domain of CSPα. However, the functional effects are currently unknown.

CSPα is a cytosolic and membrane-bound cochaperone^68^ that exhibits chaperone and cochaperone activity independent of its ability to bind membranes^69^. Most CSPα is membrane-bound due to CSD palmitoylation^62,63^. However, there is also a significant soluble fraction of CSPα in the human brain^69^, whose function is still unknown. Membrane-bound CSPα regulates secretory pathways in various cell types. In neurons, CSPα is a pre-synaptic protein that controls the folding and trafficking of SNAP-25 and dynamin by stimulating the ATPase activity of both HSC70/HSPA8 and HSP1A/HSP70^65,70–72^. CSPα knockout (KO) mice develop progressive sensorimotor defects as early as postnatal day 21, and exhibit desynchronized synaptic transmission and early death at approximately 2–3 months^34^. CSPα has been repeatedly demonstrated as essential for synaptic function pre- and postsynapse^73^. Single-cell transcriptomic data from 24-day-old CSPα KO mice revealed significant downregulation of several pathways associated with synaptic maintenance, including synapse organization (*Homer1, Ctnna2, Unc13a, Nrxn2, Prrt1, Arc, Grid2, Atp2b*), dendrite development (*Map1b, Fat3, Camk2a, Ephb1, Map6, Shank1, Numb*), regulation of synapse organization (*Asic2, Homer1, Ctnna2, Arc, Grid2, Lingo2, Etv5, Ephb1*), regulation of synapse structure (*Asic2, Homer1, Ctnna2, Ntrk2, Lingo2, Hspa8*), and postsynapse organization (*Homer 1, Arc, Grid2, Rph3a, Etv5, Ephb1, Dlg4, Sptbn2, Hspa8*)^74^. Furthermore, CSPα deficiency also results in an altered glial signature in microglia and astrocytes where most DEG are downregulated. CSPα deficiency resulted in an altered immune transcriptomic response and increased microglial activation; microglial changes also affected the robo-split and actin polymerization pathways, which are involved in synaptic maintenance^74^. In addition, CSPα deficient mice also demonstrated upregulated small GTPase and Ras protein signal transduction and an increased probability of Nrxn1-Nlgn1 interaction and communication in astrocytes^74^. CSPα is also prominently expressed in pancreatic β cells, adrenal chromaffin cells, and anterior pituitary cells. However, whether degenerative changes in these cells contribute to the phenotype of CSPα KO mice is unknown^34^. CSPα deficiency affects SNAP-25 levels and the soluble NSF attachment protein receptor (SNARE)-complex assembly, promoting vesicle fusion and fission^75^. A comparison between the synaptic proteome of wild-type and CSPα KO mice at postnatal day 28 revealed 37 proteins, including unfolded protein chaperone binding (Hsp90aa1, Hsp90ab1, Hspa5, Hsph1, Hspa8, Stip1, Hspa4l, St13, Cct5), SNARE binding (Snap-25, Cplx1, Nsf, Cacnb4), endocytic (Dnm1, Necap1), cytoskeletal (Dpysl4, Sept3, 5, 6, 7, Basp1, Dpysl2), signaling (Dbi, Ak1, Pp1cc, Dlg4, Pafah1b1, Vsnl1, Wdr7, Iqsec1), as well as other (Sec24b, Plp1, Mbp, Dynll2, Snca, Sncb) pathways^76^. A synapse-targeted approach searching for CSPα interaction partners identified at least 42 (Agfg1, Ap2a1, Ap2b1, Ap2s1, Cadm1, Cadps, Cask, Efnb1, Erc1, Fcho1, Grm8, Lin7a, Lin7c, Necap1, Nrxn3, Numb, Ppfia1, Ppfia4, Rabgef1, Scamp1, Scamp5, Sh3gl3, Slc17a7, Slc17a8, Slc18a3, Slc32a1, Snap29, Snap91, Snca, Stx12, Stx3, Stx6, Stx7, Syp, Syt1, Syt5, Vamp1, Vamp2, Vamp3, Vamp4, Zdhhc17 and Zfyve20) prominently membrane-associated proteins or containing membrane-spanning domains^76^. Interestingly, CSPα mutations (L116Δ and L115R) reduced the interaction with membrane proteins but increased the interaction with nonmembrane proteins (Agfg1, Ap2b1, Erc1, Rabgef1, Scamp1, Snap91, Zdhhc17)^77^. It is known that membrane-bound CSPα plays different roles in different cell types and subcellular compartments. However, in the current body of literature, only a limited subset of CSPα interactors have been identified and thoroughly studied. This is mainly due to these previous studies primarily focusing on pathways only specific to synaptic maintenance.

**Fig. 3:**
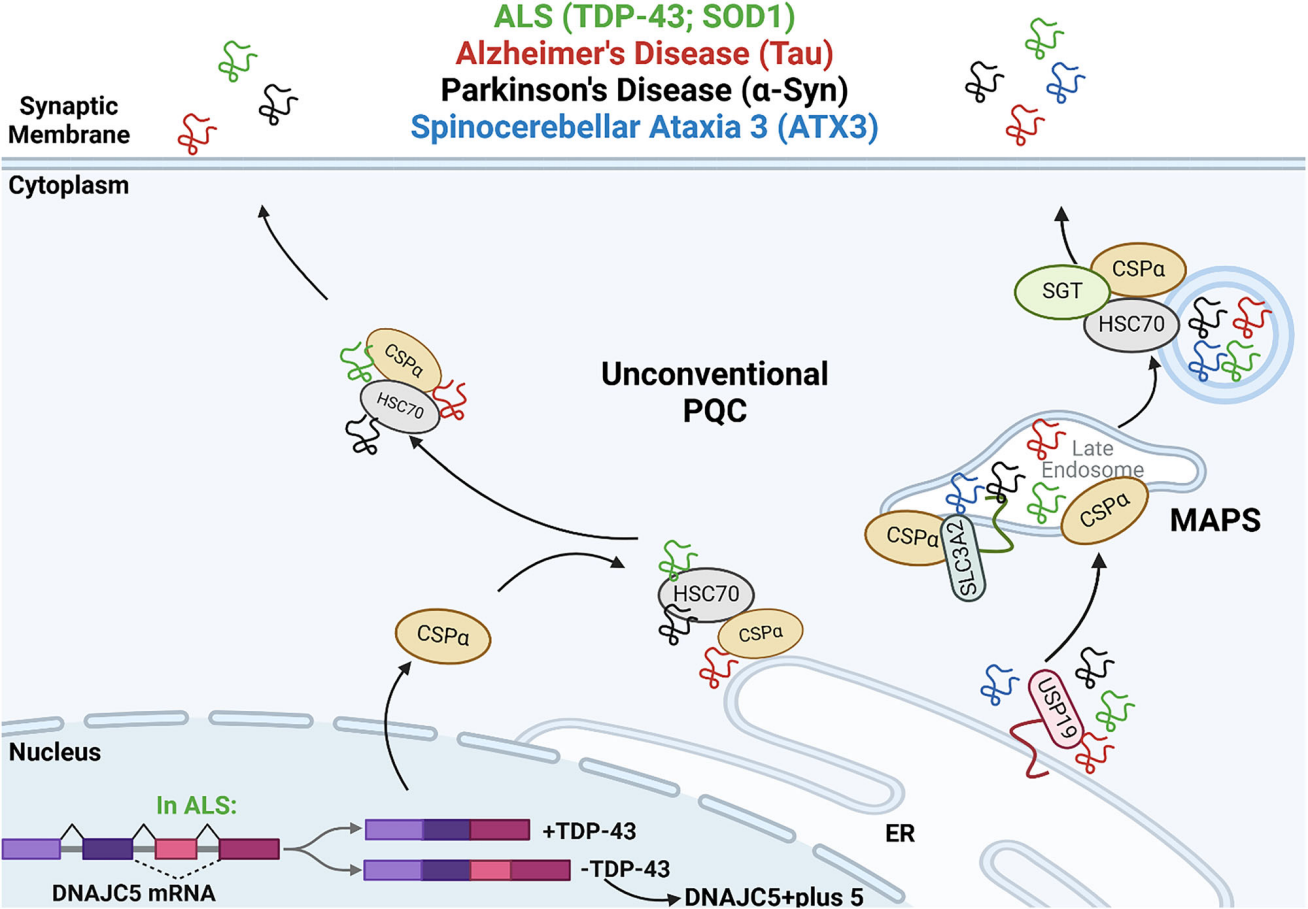
The role of CSPα in the intracellular trafficking of misfolded disease-associated proteins in common dementia. Misfolded proteins are recruited to the ER by USP19. CSPα then facilitates secretion either through an unconventional protein quality control pathway^78^ or MAPS^58^ of the ALS-associated TDP-43 (green) and SOD1 (green), Alzheimer’s-associated tau (red), Parkinson’s-associated α-Syn (black), or spinocerebellar ataxia 3-associated ATX3 (blue), via a SLC3A2-dependent pathway. In addition, in some instances of ALS, TDP-43 mutation leads to alternative splicing of *DNAJC5*, including an extra cryptic exon^67,141^. This effect on gene overall gene function has yet to be fully explored.

A common feature among neurodegenerative diseases is the transsynaptic spread of aggregate-prone proteins in a prion-like manner^8^. In vitro studies (M17 neuroblastoma and HEK293T cells) have shown that CSPα is crucial for the SNAP-23-mediated secretion of aggregate-prone proteins, including tau, α-synuclein, and TDP-43^78^. This secretion of misfolded cytosolic proteins has been proposed to proceed through an unconventional PQC pathway or a recently described secretion pathway termed USP19-regulated misfolded-associated protein secretion (MAPS)^58,79^ (Fig. 3). In MAPS, misfolded proteins are recruited by USP19 to the surface of the ER^58^ where CSPα facilitates the secretion of proteins^61^. Based on the established role of CSPα in the presynaptic compartment and the recently demonstrated participation in secretory pathways, we propose that neuronal CSPα facilitates the secretion of aggregate-prone proteins into the extracellular space. Thus, CSPα may promote cellular proteostasis by removing locally misfolded aggregates without placing an undue proteotoxic burden on the degradative organelles of an individual cell^80^. However, this rapid purging of misfolded proteins could also facilitate the proteopathic spread of these aggregates to neighboring cells and serve as a seed for further aggregation^8^.

## Human genetics and a paradigm shift in the role of CSPα in endolysosomes

Autosomal dominant-adult neuronal ceroid lipofuscinosis (AD-ANCL), or Parry’s disease, is a rare lysosomal storage disorder and neurodegenerative disease characterized by progressive cognitive decline, loss of motor function, visual impairment, dementia and death^81,82^. In a family with numerous members who had AD-ANCL and early dementia, exome sequencing linked the disease to two mutations within the CSD of CSPα: L115R and L116Δ^81^. This discovery of AD-ANCL-causing mutations in CSPα opened a new area of research into the function of CSPα at the endolysosome^81,83,84^. The role of CSPβ and CSPγ as modifiers of the ANCL phenotype is unknown.

More recently, additional DNAJC5 mutants have been reported, including p.L116R, p.C128Y, and p.C124_C133Dup have been reported^85–87^. All the ANCL-causing mutations are located within the CSD of CSPα, all the mutations except p.L116R result in high-molecular-weight aggregates (HMWAs), and all affect CSPα palmitoylation and subcellular sorting (Fig. 2). Mutant CSPα HMWAs are induced and maintained by palmitoylation^63,88^. We and others have shown that chemical depalmitoylation solubilized the CSPα HMWAs in vitro and in vivo^63,83,88,89^.

Mutant CSPα (L116Δ and L115R) reduces monomer palmitoylation^62,63^ and likely increases the soluble mutant CSPα fraction^69,89^. These two mutants also increase the affinity for the palmitoyltransferase Zdhhc17^77^. CSPα is a highly palmitoylated protein with a long half-life. PPT1 depalmitoylates proteins inside the lysosome before they are degraded. We found a significant increase in the PPT1 activity in the brain and ANCL-derived cells^89^. All these findings agree with the reported shorter half-life than that of wild-type CSPα due to lysosomal degradation of those mutants^61,89^, which could explain the significant reduction (95%) in soluble CSPα in ANCL patients^69^.

We reported the histological features of the brain of an asymptomatic individual carrying a *DNAJC5* mutation, which shows typical autofluorescent storage material (AFSM) in neurons with minimal neuroinflammation but no evidence of massive synaptic loss or cerebral atrophy^69^. We detected no significant differences in CSPα or synaptophysin levels in the neuropil from an asymptomatic mutation carrier^69^. Thus, it is likely that early changes during ANCL progression stem from CSPα aggregation affecting endolysosomal fusion events, resulting in the formation of “vesicular traffic jams” and the AFSM along with the subsequent enlargement of the cell body, which precedes synaptic dysfunction, synaptic loss and, eventually, cell death.

Postmortem analysis of terminal ANCL cases revealed significant neuronal loss, extensive synaptic degeneration in multiple cortices (frontal, parietal, and temporal), and abundant accumulation of AFSM in all the tested cells, including neurons and cells from the skin^90–93^. We showed that terminal ANCL patients exhibit lysosomal dysfunction, including increased levels of LAMP1, LAMP2 and V-ATPase B1/2 protein, along with significant secondary elevations in the activity of the lysosomal enzymes PPT1, β-glucuronidase (β-gluc), and β-hexosaminidase (β-Hexa) in multiple brain regions^89^. These findings were independently confirmed via a proteomic approach in ANCL brains, which revealed significant changes in lysosomal proteins rather than synaptic proteins, resulting in increased levels of 14 proteins (Ppt1, Psap, Ndufa8, Sv2a, Aldh2, Ctsd, Slc25a12, Ndufa9, Sept5, Clu, Cox7a2, Ndufa5, Slc1a3, Frem2) and decreased levels of 3 proteins (Hapln2, Cst3, Dnajc5)^64^. ANCL terminal cases exhibited significantly reduced levels (50%) of membrane-bound CSPα levels and the SNARE complex (SNAP-25, VAMP2, and STX1), but not of HSC70 levels^83,88,94^. Terminal ANCL brains also exhibit higher proteasome activity than healthy controls^89^. All this evidence suggests a high degree of proteotoxic stress in ANCL patients brains. In the absence of CSPα, the proteasome degrades many misfolded CSPα partners^75^. Thus, it is likely that reductions in SNARE partners result from reductions in membrane-bound and soluble CSPα and high proteasomal activity in ANCL brains.

**Fig. 4:**
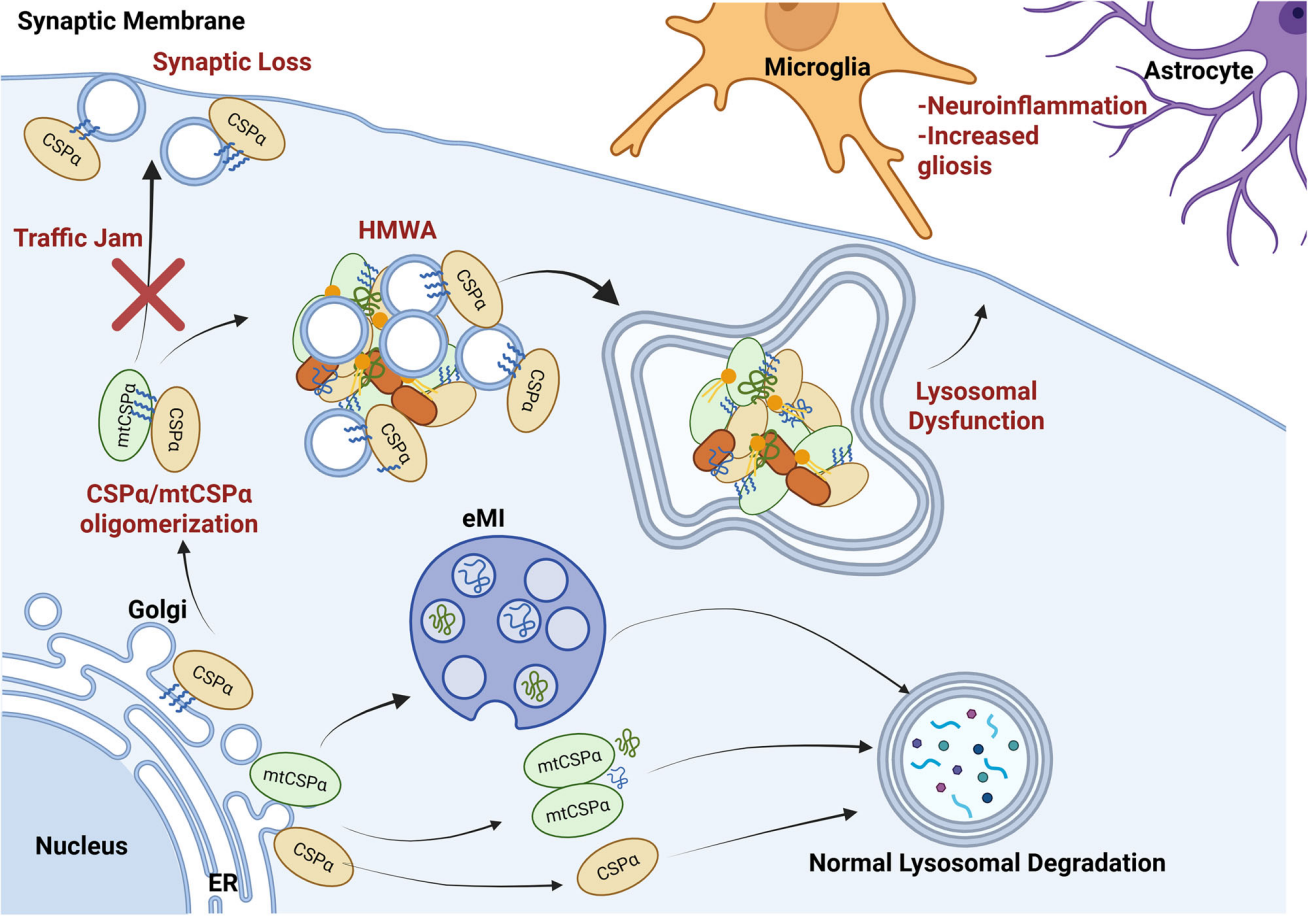
ANCL molecular pathophysiology. Depending on its palmitoylation status, CSPα regulates proteostasis by transporting misfolded proteins either to the synaptic membrane for secretion or to the lysosome for degradation. In ANCL, heterodimers of both WT and mutant CSPα disrupt these pathways, causing aggregation, endolysosomal dysfunction, and traffic jams, ultimately leading to synaptic loss and increased gliosis (sequence shown in red). Heterodimers of CSPα/mtCSPα lead to aggregation and endolysosomal dysfunction before resulting in synaptic loss and increased gliosis (sequence shown in red). Mutant CSPα (mtCSPα) aids in the endolysosomal loading and transport of misfolded proteins, which are then degraded by the lysosome. The lysosome also degrades both WT and mutant CSPα monomers or homodimers in the absence of aggregation.

Palmitoylated CSPα binds secretory vesicle membranes in various cell types. In neurons, CSPα binds to synaptic vesicles. However, a significant proportion of membrane-bound CSPα also localizes to the endolysosome in the perinuclear compartment, with some at the cell surface^30,89,95,96^. In three different cell types, including a neuron-like cell line and primary cortical neurons, we showed that endogenous CSPα colocalizes with lysosomal markers and is enriched in lysosomal fractions^89^. CSPα has been found in both autophagosomes^97^ and lysosome-enriched fractions independently via proteomic approaches^98–101^. In CSPα-deficient mice, autophagic dysfunction is observed at the synaptic terminal, as shown by increased autophagosomes found at the presynapse of inhibitory neurons and increased colocalization of the autophagic protein Atg9a and the presynaptic marker amphiphysin^74^. Additional autophagic markers, such as Atg16l1, Atg10, and Becn1, are also upregulated in inhibitory neurons^74^. In both in vitro and *Drosophila melanogaster* models, whether CSPα is trafficked to the synapse or the endolysosome depends on its interaction with SLC3A2 via its linker domain^61^. Knockdown of SLC3A2 resulted in the redirection of CSPα localization to the endolysosome (peri-nuclear LAMP1-positive). SLC3A2 is deemed indispensable for the participation of CSPα in MAPS^61^. CSPα overexpression alone affects the lysosomal pH and increases the levels of secreted lysosomal enzymes and the fusion of lysosomes with the plasma membrane^89^, suggesting an increase in lysosomal exocytosis. In addition, we demonstrated that, unlike the UPS, the lysosome mainly degrades CSPα in response to the activation of the ALP^89^. We showed that the endolysosomal dysfunction in ANCL patient-derived cells is a direct consequence of CSPα in the endolysosome, affecting the levels of lysosomal proteins (LAMP1, LAMP2, and V-ATPase B1/2), Rab7 and synaptosome-associated protein of 23 kDa (SNAP23)^89^. The SNARE complexes mediate fusion events between endosomes, phagosomes, autophagosomes, or plasma membranes and lysosomes. The interactome of CSPα includes syntaxin-7, syntaxin-8, vesicle-associated membrane protein-8 (VAMP8), VAMP7, SNAP23, synaptotagmin-VII and Rab3a, which form trans-SNARE complexes for lysosome fusion^89^. Thus, CSPα could play a role in lysosome-membrane fusion events, including lysosome exocytosis (Fig. 4).

All ANCL-causing mutations promote the oligomerization of CSPα^64,69,81,88,89,102^ further promoting heterocomplex formation between the wild-type and the mutant proteins (Fig. 4). These mutations disrupt chaperone activity and alter palmitoylation, resulting in missorting and reducing membrane binding ability^81,83,84,103,104^. Disruption of CSPα palmitoylation increases the amount of soluble mutant CSPα and facilitates its interaction with normal soluble CSPα to form the HMWAs, which ultimately could reduce soluble CSPα by sequestering it into aggregates along with many of its partners, suggesting that the pathogenic mechanism of CSPα acts as a dominant-negative (DN) mutation. The formation of heterodimers between mutant and normal CSPα is necessary to induce lysosomal dysfunction^89^. Mutant CSPα alone is unable to cause lysosomal dysfunction^89^, likely because it maintains the CSPα microautophagy- stimulating activity of misfolded CSPα partners but abolishes CSPα-MAPS secretion, favoring intracellular accumulation^61^. CSPα palmitoylation is necessary to redirect these degradation-resistant HMWAs away from the endolysosome^61^. We demonstrated that combining a depalmitoylating small molecule (N-tert-butyl-hydroxylamine) and activating the autophagy-lysosomal pathway (serum withdrawal and ATP-competitive mTOR kinase inhibitor, Torin 1) was enough to facilitate the degradation of HMWAs in ANCL-derived cells^89^. We propose a sequential cascade of pathological events in ANCL, triggered by CSPα aggregation into HMWAs, followed by endolysosomal dysfunction and vesicle “traffic jams,” leading to synaptic dysfunction and synaptic and neuronal loss, followed by microgliosis (Fig. 4).

## The role of CSPα in common neurodegenerative diseases and dementia

A growing body of evidence also implicates CSPα in several neurodegenerative diseases characterized by dementia^105^. In most of the conversion of native proteins into pathogenic conformations, the cell-to-cell propagation and spreading throughout brain models follow this pattern: an intracellular and misfolded pathological protein forms aggregates that are released into the extracellular space and taken up by neighboring cells where they interact with the endogenous protein, developing new intracellular aggregates in the recipient cells^8^. In this section, we delve further in-depth to explain the potential role of CSPα in several steps of the pathological spreading process and how CSPα-dependent proteostatic failure may lead to worsening instances of dementia in patients.

### Alzheimer disease

AD is the most prevalent form of dementia and is characterized by amyloid (Aβ) plaques and neurofibrillary tangles (NFTs, Tau deposits) in the brain, accompanied by neuroinflammation, myelination changes, synaptic dysfunction and loss, gliosis, and neuronal death^106^. Synaptic loss is considered a strong and accurate correlate for disease severity in AD. Evidence from several animal models of AD pathology reveals dysfunctional synaptic signaling^107–109^. Analysis of the synaptic proteome from human AD patients revealed a significant reduction in pathways associated with synaptic function^110^. CSPα expression is reduced in the hippocampus and superior temporal gyrus^110,111^, and medial prefrontal cortex^76^ in AD patients. A reduction in CSPα in the hippocampus occurs even earlier than a reduction in synaptophysin levels, suggesting that decreased CSPα levels may be an even earlier and more sensitive marker for synaptic degeneration than other synaptic markers^111,112^. However, CSPα is also elevated in the cerebellum in AD patients, an area unaffected by the disease^111^. Recently, it was reported that AD brains exhibit amorphous deposits of CSPα that form in the outer periphery of amyloid plaques^112^. CSPα has been demonstrated to be co-secreted along with identified substrates of the MAPS pathway^58^, so it may be possible that Aβ is another neurodegenerative aggregate-prone peptide whose secretion is facilitated by CSPα, whether by MAPs or some other yet-to-be-determined pathway. However, more work will need to be done to demonstrate this adequately. DNAJB1, a DnaJ family member of CSPα, has been shown to bind in vitro to tau and Aβ aggregation along with Aβ fibril formation^29^. Aβ plaques also follow a predictable spatiotemporal pattern of propagation that affects connected areas of the brain during AD progression^8^. CSPα also facilitates lysosomal secretion, another PQC process that promotes the exocytosis of stressed lysosomes from the cell. CSPα likely enables the secretion of Aβ from neurons under proteostasis stress to restore and maintain homeostasis^89^. However, there is still little evidence to directly demonstrate that CSPα facilitates the secretion of Aβ.

The other main component of the pathological features of AD is the intracellular accumulation of tau. Tau converts into β-sheet-rich conformations that underlie the pathology of ~25 neurodegenerative diseases known as tauopathies, including AD and frontotemporal lobar degeneration (FTLD)^113^. Tau has a highly stereotyped pattern of spreading through multiple brain regions^114^. Although the mechanisms of tau spreading are not fully understood, the autophagy lysosomal pathway plays a pivotal role in both degradation and propagation^115^. Tau can be targeted for degradation by the lysosome through various autophagic pathways, such as chaperone-mediated autophagy (CMA), endosomal microautophagy, macroautophagy, or autophagy-independent endolysosomal degradation^115^. Alternatively, neurons may secrete tau through extracellular vesicles to relieve proteotoxic stress. This secreted tau can then be uptake by neighboring cells such as other neurons, microglia, and astrocytes. Depending on the recipient cell, the pathway of secretion/uptake, and the tau secreted/uptaken forms, this can further tau spreading and propagation throughout the brain^115^. Failure in any of these processes has been linked to the development of tauopathies. In addition, several genes involved in autophagy and lysosomal pathways, such as BIN1, PICALM, and GRN, have significant evidence implicating them in tau pathology^115^. Various groups have demonstrated that CSPα promotes the exocytosis of MAPT (tau) secretion via MAPS, exosomes, or in a SNAP23-mediated unconventional pathway^58,78,80^. Molecular chaperones regulate normal tau function and aggregation in multiple diseases^116^. In a mouse model of tauopathy, CSPα levels were inversely correlated with pathological burden in the hippocampus and cerebellum, even without neuronal loss^111^. However, it remains unknown which tau species are released via the CSPα-mediated pathway and whether they have seed activity to promote the aggregation of native tau in neighboring cells.

### α-Synucleinopathies

The accumulation of α-synuclein in Lewy bodies (LBs) and Lewy neurites (LNs) across multiple brain regions from the brainstem to the neocortex^117^ is a defining feature of numerous synucleinopathies such as PD, dementia with Lewy body (DLB), and diffuse Lewy body disease^118,119^. α-synuclein aggregates at synapses in the brain during the early stages of disease^11^. Aggregation of α-synuclein is further regulated by *DNAJB1* and *DNAJB10* in in vitro and *C. elegans* models, respectively ^29^. The spread of α-synuclein throughout the brain mediates the spatiotemporal pathology^120^. However, the mechanisms underlying cell-to-cell transmission are unknown. CSPα facilitates the secretion of α-synuclein via MAPS, exosomes or a SNAP23-mediated unconventional pathway^78,80,121^. However, the mode for this secretion is not entirely clear. CSPα loads α-synuclein into the lumen of late endosomes^121^ via VPS4-dependent ESCRT-dependent microautophagy and then secretes it via SLC3A2-assisted MAPS^61^. Palmitoylated CSPα oligomers mediate an alternative route of α-synuclein secretion^121^.

The relationship between CSPα and α-synuclein is quite complex. The overexpression of transgenic α-synuclein reversed the synaptic loss and cognitive decline and increased the survival of CSPα KO mice, and α-syn assists CSPα with proper SNARE complex assembly, underscoring the importance of both proteins to synaptic health^72,122,123^. In Drosophila, heterozygous loss-of-function of DNAJC5 enhances α-synuclein pathology^124^. In contrast, CSPα overexpression reduced α-synuclein aggregation, increased the levels of α-synuclein monomers, and improved vesicle cycling and dopamine secretion^11^. Taken together, these findings suggest that CSPα regulates the secretion of α-synuclein. However, the mechanism and the role of CSPα in neurodegenerative diseases associated with the accumulation of α-synuclein are still unknown.

### Huntington’s disease

HD is a dominantly inherited movement disorder first described in 1872 by George Huntington and presents itself through jerky and involuntary (referred to as choreiform) movements^125,126^. HD prototypically presents itself through the progressive basal ganglia and whole brain atrophy along with concurrent cognitive deterioration^127^. Notably, dementia is also considered a prominent symptom of HD and can present itself even earlier than the first motor symptom^128^. However, while dementia in HD patients has long been assessed under the same criteria as AD, it has since been understood that HD-related dementia presents itself differently than AD^129,130^. While dementia in AD is more characterized by memory loss, dementia observed in HD is characterized by a significant loss of other areas of cognition (i.e., attention, processing speed, and executive functions)^130^. HD is caused by CAG trinucleotide repeat expansion in the HTT gene encoding polyglutamine (polyQ) repeats^125^. Polyglutamine tract expansion results in the formation of HTT inclusion bodies, one of the neuropathological hallmarks of HD. Mutant HTT (mHTT) can disrupt transcription, have deleterious effects on immune and mitochondrial function, and can be aberrantly modified posttranslationally, driving aggregation^125,126^. Molecular chaperones have been consistently identified as modifiers of polyQ aggregation^131^. In Drosophila, dHDJ1 has been shown to increase soluble and aggregated polyQ, yet reduce htt toxicity and eye degeneration, while in cell models, DNAJB6b and DNAJB8 reduce polyQ aggregation^29^. Similarly, CSPα has also been implicated in the formation of htt inclusions in a palmitoylation-dependent manner^132^. While CSPα is palmitoylated by HIP14/DHHC17^64^, blocking this palmitoylation has increased htt inclusion formation^132^. CSPα participates in mHTT extracellular secretion via a SNAP23-mediated pathway^78^ or via 180–240 nm and 10–30 µm extracellular vesicles^80,133^. Intriguingly, mHTT binds and sequesters CSPα, specifically when the polyglutamine is present in mHTT but not in normal HTT^134^. It is unclear whether the binding CSPα to mHTT increases its accumulation or favors its disaggregation and facilitates its secretion from the cell, which plays a role in its prion-like propagation. Notably, Csp-null Drosophila exhibit paralytic and uncoordinated sluggish movements, spasmic jumping, intense shaking, and a reduced lifespan^52^.

### Amyotrophic lateral sclerosis

ALS, or Lou Gehrig’s disease, is characterized by neuron loss of upper and lower motor neurons and progressive paralysis, suggesting a pathomechanism based on spreading through neuronal connectivity^135,136^. Many ALS patients develop the cognitive and behavioral deficits observed in frontotemporal dementia (FTD), which are generally termed amyotrophic lateral sclerosis-frontotemporal spectrum disorder (ALS-FTSD)^137^. The pathological aggregation of TDP-43 is found in nearly all ALS cases and almost half of all FTLD cases^138^. In addition, SOD1 also forms accumulations in ALS, with ~20% of familial ALS cases and ~2% of sporadic ALS cases presenting SOD1 mutation^139^. Both TDP-43 and SOD1 have been suggested to be MAPS substrates, with overexpression of CSPα and USP19 promoting their extracellular secretion in in vitro studies^58^. Both are also regulated by multiple DnaJ protein family members, with DNAJB1 reducing SOD1 aggregation and DNAJB2 reducing both SOD1 and TDP-43 aggregation^29^.

Interestingly, while several mutations in TDP-43 have been identified to cause both ALS and FTLD, these mutations are only present in a small percentage of cases for both diseases^138^. TDP-43 is an RNA/DNA-binding protein (RBP) that facilitates gene transcription, regulates mRNA stability and translation, and, more importantly, for this review, regulates mRNA splicing^140^. TDP-43 serves as a splicing suppressor of nonconserved cryptic exons^141,142^ for several genes. This effect is dependent on the O-glycosylation of TDP-43 by O-linked N-acetylglucosamine transferase^66^. However, its misfolding and abnormal phosphorylation result in its mislocalization to the cytosol over the nucleus, aggregating and disrupting many of these important molecular functions^143,144^. This cytosolic accumulation can have several cytotoxic consequences, sequestering essential cellular components, generating oxidative species, and inhibiting the proteasome^135^. However, most notably, by aggregating within the cytosol, TDP-43 is depleted in the nucleus, leading to abnormal splicing of several genes^141,142^. However, seed-competent TDP-43 has been found in exosomes in the cerebrospinal fluid of ALS patients^145^.

Many details of the spread of TDP-43 are not well understood. TDP-43 has been consistently shown to alternatively splice the mRNA transcript for DNAJC5 in both mouse and human cell lines^66,67,141,146^. Notably, when TDP-43 was knocked down through ASO-transduction in mice, the splicing of Dnajc5 was altered to include an additional exon, resulting in a larger transcript and protein product^141^. The same phenomenon is observed in human-derived induced pluripotent stem cells carrying the TDP-43^K263E^ mutation^67^. The effect of this added exon on CSPα function remains to be understood. Interestingly, CSPα facilitates the secretion of TDP-43 in vitro^78^. However, additional studies are needed to fully understand the bidirectional relationship between TDP-43 and CSPα and their roles in human diseases.

### Prion disease models

Prion diseases are a group of transmissible neurodegenerative diseases^147^. It has been suggested that synapses are the hubs of a convergent mechanism for different protein misfolding seeds, including prion proteins. A reduction in hippocampal CSPα (50%) was found in C57BL/6 mice injected with murine-modified scrapie (ME7), which has been demonstrated to induce a model of prion disease in vivo^148^. This finding could be part of a specific synaptic protein loss in this model since there was a significant reduction in the levels of two other synaptic vesicle proteins (synaptophysin and synaptobrevin). However, synaptotagmin-1 levels remain unchanged^148^. In a separate study, a decrease in CSPα levels in ME7-injected mice did not exacerbate the neurodegenerative changes^149^. Thus, whether the mechanism underlying the selective reduction of some synaptic vesicle proteins in prion mouse models includes CSPα or whether increasing CSPα levels could modify the synaptic changes is unclear.

## CSPα as a therapeutic target for human diseases in which proteostasis fails

CSPα plays roles in the endolysosome and the synapse, controlling the secretion of important proteins implicated in neurodegeneration. There is an age-dependent reduction in DNAJC5 transcript and protein levels in the brain and cerebellum of healthy individuals^111^. CSPα reduction in the degenerating regions of AD patients has been proposed to serve as a promising early indicator of reduced synaptic function and disease^110–112^. Therefore, we consider strategies to increase the CSPα levels in the development of potential therapeutics against neurodegeneration.

CSPα activity can be modulated through pharmacological interventions. The plant-derived flavonoid quercetin promotes CSPα dimerization and affects synaptic transmission^121,150^. Iron chelators reduce CSPα oligomerization and partially rescue SNARE complex formation in mouse primary cortical neurons expressing ANCL-causing mutations^104^. Lithium is a commonly used treatment for psychiatric diseases and has been shown to increase CSPα expression in the cell body and dendritic boutons both in vitro (PC12 cells) and in vivo (in the brains of rats)^151^. Lithium chloride improves cognitive impairment and appears to restore and stabilize SNARE complex proteins (CSPα, SNAP-25, and syntaxin-1), which are reduced following traumatic brain injury^152^. Additionally, benzoquinone, ansamycin, and geldanamycin induce an HSR, increasing the CSPα and HSP70 levels and inhibiting the aggregation of mHTT in vitro^153^. Antidepressants and amphetamines also increase CSPα levels in the rodent brain^154,155^. Chronic morphine treatment downregulates CSPα and Hsc70 expression in the rat striatum^156^. Recently, CSPα was reported to potentially interact with mTOR to maintain brain health^157^. We previously demonstrated that activating the ALP had a beneficial effect on ANCL-derived cells. However, whether these pharmacological modulations of the CSPα levels have functional consequences remains to be determined. In addition, many pharmacological small molecules that modulate CSPα have a broad spectrum of targets, making it challenging to select agents for specific neurodegenerative disease applications.

Adeno-associated virus (AAV)-mediated gene therapy has emerged as an effective delivery strategy for various human disorders^158^, including AD, PD, and HD^159,160^. Until 2023, the U.S. Food and Drug Administration (FDA) has approved Kymriah, Yescarta, Luxturna and Zolgensma^158^. AAV serotype 2 is commonly used for the treatment of brain disorders because of its persistent expression in postmitotic cells and favorable safety profile^161,162^. Approximately 200 clinical trials involving thousands of participants have used AAVs in humans^163^. The widespread pathology of neurodegenerative diseases is considered unamenable to gene therapy. Human genetic studies have identified potential targets for preclinical and clinical applications^164,165^. There are increasing numbers of clinical trials of gene therapy for brain disorders^159^. Initial studies in Drosophila revealed that the overexpression of CSPα regulates secretory pathways that mediate fusion events at the plasma membrane or contribute to the secretion of growth factors or regulatory proteins that favor synaptic formation or stabilization^166^. Recently, AAV-mediated overexpression of CSPα in mice was shown to reduce α-synuclein-induced neurodegeneration^11^ and rescue lysosomal and synaptic deficits in a mouse model of lysosomal storage disease^167^. Thus, AAV-mediated delivery of CSPα under neurodegenerative conditions with a reduction in synaptic integrity could improve the degradation of aggregate-prone proteins and maintain a proper synaptic function.

## Conclusions

In this review, we discuss the evidence demonstrating the crucial role of CSPα in maintaining both endolysosomal and synaptic integrity and its specific role in the pathogenesis of several neurodegenerative dementias. However, CSPα appears to play a multi-faceted role in both neurodegeneration and neuroprotection. *DNAJC5* mutations in humans and loss-of-function in model organisms cause neurodegeneration, which leads to the notion that CSPα is “neuroprotective”. Furthermore, overexpression of CSPα rescues neurodegenerative phenotypes in mouse models of human diseases. Cautiously, increasing evidence suggests that CSPα interacts directly or indirectly with aggregate-prone proteins relevant to common neurodegenerative diseases and favors their secretion, suggesting that CSPα can potentially exacerbate neurodegeneration. Additional studies are needed to determine the molecular pathways underlying the compromised cellular CSPα-neuroprotective pathways in common neurodegenerative disorders. Furthermore, further studies will be necessary to examine the possibility of leveraging the neuroprotective potential of CSPα to maintain proteostasis in preventing and treating dementia.

## Data Availability

No datasets were generated or analyzed during the current study.
